# Molecular Simulation and Theoretical Analysis of Slide-Ring Gels under Biaxial Deformation

**DOI:** 10.3390/gels7030129

**Published:** 2021-08-29

**Authors:** Kotaro Tanahashi, Tsuyoshi Koga

**Affiliations:** Department of Polymer Chemistry, Kyoto University, Kyoto 615-8510, Japan; tanahashi@phys.polym.kyoto-u.ac.jp

**Keywords:** slide-ring gels, biaxial deformation, molecular simulation, slip-link model, theory

## Abstract

Slide-ring (SR) gels, a new type of gels that have cross-links moving along the chains, are known to have unique mechanical characteristics. In the case of biaxial deformations, it has been experimentally shown that the stress–strain (S–S) relationships of SR gels can be well described by the neo-Hookean (NH) model. This behavior is quite different from that of conventional chemical gels, where the S–S curves deviate from the NH model. To understand the molecular mechanism of such peculiar elastic properties of SR gels, we studied the effects of movable cross-links by using molecular simulations and theoretical analysis. We calculate the S–S relationships in biaxial deformation for two types of models: slip model, where the cross-links can slide along chains representing SR gels, and non-slip model, which corresponds to conventional chemical gels. In the theoretical analysis, we calculate the S–S relationships by using the models with the Gaussian and the Langevin chains to investigate the nonlinear stretching effect of the chain in the slip and non-slip models. As a result, we found that the peculiar elastic behaviors of SR gels in biaxial deformations are well explained by the effect of movable cross-links suppressing the nonlinear stretching of the chain.

## 1. Introduction

Gels are, traditionally, classified into two types: chemical gels and physical gels, depending on the strength of the bond energy of cross-links. The cross-links of chemical gels are formed by chemical covalent bonds, which can not be broken by thermal motion of molecules. In physical gels, the network is cross-linked by non-covalent bonds such as hydrogen bonds or ionic interaction. However, a novel type of gels, called slide-ring (SR) gels, belonging to neither of such types, were developed by Ito’s group [1]. SR gels are prepared by cross-linking polyrotaxane (PR) consisting of many cyclic molecules (cyclodextrin: CD) threaded on a main chain end-capped with bulky groups [2,3]. The figure-of-eight cross-links in SR gel can slide along the chains and act like pulleys to vary the network structure in response to imposed deformation. By this so-called pulley effect, the stress on the network under deformation can be effectively relaxed [4]. The mechanical properties of SR gels are drastically different from those of the conventional chemical gels; thus, a great deal of attention has been given to SR gels from not only scientific but also engineering fields.

The elastic properties of SR gels have been studied extensively by experiments [5,6,7,8,9,10,11], theories [12,13,14] and molecular simulations [15,16]. The unique elastic features of SR gels have been studied in the measurement of stretching-driven swelling [5]. It was shown that the equilibrium Poisson’s ratio in the stretching-driven swelling is dependent of the strain in SR gels, whereas that of conventional chemical gels does not depend on the strain. This behavior can be explained by the relaxation of the orientational anisotropy of the network induced by the movement of cross-links. It was also reported that SR gels exhibit characteristic relaxation time in viscoelastic measurements [6,7,8].

From the theoretical viewpoint, cross-links which can slide along the chain were originally introduced as slip-links to describe the mechanical effect of chain entanglements in chemical gels [12,13]. This theory, known as slip-link theory, successfully explains the nonlinearity of the S–S relationship of the network with chain entanglements. The network with slip-links has also been theoretically studied with different models [14]. One of the authors of this paper has studied the mechanical properties and structure of the network with tri-functional sliding junctions under uniaxial deformations, by using Brownian dynamics simulations [15].

Recently, biaxial deformations that have two independent stretching axes, have been used to investigate the elastic properties of gels [17,18,19,20,21,22]. In uniaxial deformations, the stress is measured along only one direction. As a result, theoretical models for rubber elasticity can easily fit the experimental data in uniaxial deformation, which makes it difficult to assess the model validity. In biaxial deformations, two independent stretching ratios: the stretch ratio in the *x*-direction and *y*-direction, cover all possible deformations. This gives us more information about the S–S relationships to assess the validity of the theoretical models comprehensively [18]. For example, the S–S relationships of chemical gels in uniaxial deformation are described well by the neo-Hookean (NH) model [23], while, in the case of biaxial deformation, the S–S curves deviate upward from those of NH model.

Contrary to such previous results, a recent study [20] shows that the S–S relationships of SR gels in both uniaxial and biaxial deformations are very close to those of NH model. This result is interesting since the NH model which has been used to describe the elasticity of conventional chemical gels, failed to fit the S–S curves of chemical gels in biaxial deformation. On the other hand, the NH model successfully fits the S–S curves of SR gels whose network structure is drastically different from chemical gels, in both uniaxial and biaxial deformations. The molecular mechanism of this peculiar nonlinear elasticity of SR gels has not been elucidated. The purpose of this paper is to reveal the molecular mechanism which characterizes the S–S relationship of the network with slip-links by using Brownian dynamics simulations and theoretical analysis.

First, we calculate the S–S relationships of the network with slip-links (slip model) and the network with fixed cross-links (non-slip model) in two step biaxial deformation by using molecular simulations, in order to confirm that the results by the simulation are consistent with those of experiments. Next, we calculate the S–S relationships of slip model and non-slip model based on the Flory–Rehner tetrahedral model to analyze the properties of the slip model in more detail. Furthermore, in the theoretical models, we compare two types of chains, the Gaussian chain and the Langevin chain, to investigate the effect by the nonlinear stretching of the chain.

## 2. Results and Discussion

### 2.1. Molecular Simulations

We employed the bead-spring model in our coarse-grained molecular dynamics simulations to calculate the S–S relationship of the slip and the non-slip models [24]. In our previous work, one of the authors has introduced tri-functional slip-links in which both ends of the polymer chain slide along the different chain backbone [15]. Here, we introduce the tetra-functional slip-links corresponding to the figure-of-eight cross-links. If one wants to model the slip-link imitating the actual structure of CD molecules, a slip-link needs to be represented by a ring consisting of several beads. However, to study many chain problems efficiently, we replaced the rings by pseudo beads representing the positions of slip-links along the primitive path of the chain.

#### 2.1.1. Model

**Figure 1:**
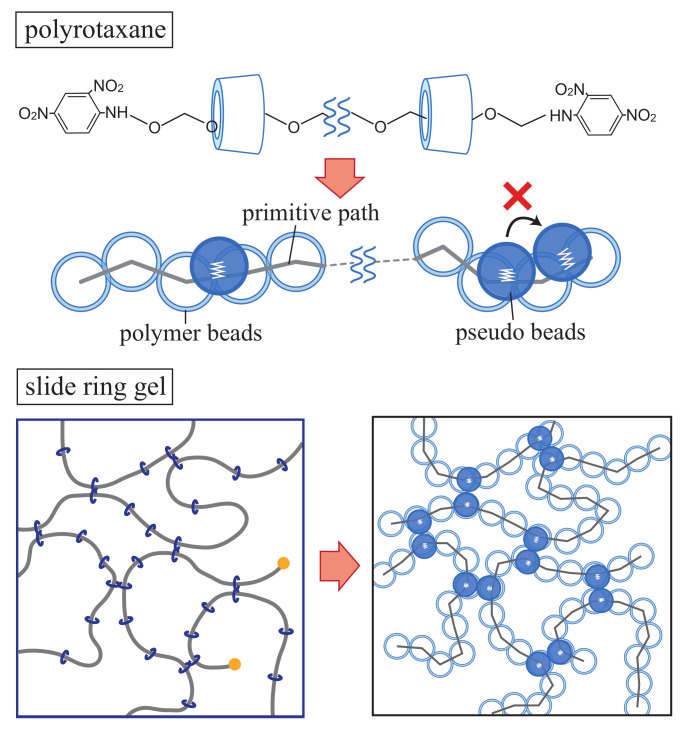
Schematic figures of the models for polyrotaxe and SR gels used in the simulation. The polymer beads are represented by outlined beads. The pseudo beads which represent the position of CD molecules are shown as filled beads.

First, we modeled the polyrotaxane by using the bead-spring model (Figure 1). A main chain is composed of successive $n_{\mathrm{PEG}}$ polymer beads connected by the nonlinear springs. Next, we introduced $n_{\mathrm{CD}}$ pseudo beads to represent the position of CD molecules. We used the parameters: $n_{\mathrm{PEG}}=20$ and $n_{\mathrm{CD}}=6$. The pseudo beads are bonded to the nearest point on the primitive path by the nonlinear spring, i.e., it can slide along the chain. Repulsive excluded volume interaction acts between pseudo beads to prevent them from passing through each other, whereas no interaction acts between a pseudo bead and a polymer bead. In our model, the pseudo beads are not allowed to leave the chain to model the actual polyrotaxane end-capped with bulky groups to confine CD molecules on the chain. By connecting the pseudo beads on different chains with the nonlinear spring, we introduced cross-links corresponding to the figure-of-eight cross-links in the SR gels. SR gels are prepared as follows in the experiments. PR is dissolved with the cross-linker in a solution. The gelation is conducted by covalently cross-linking cyclodextrins on PR chains at room temperature for a day. Finally, the gel is washed with water to stop the gelation [20]. In the simulation, we formed a network in the following way. First, we prepared the main chain with pseudo beads in the simulation box and ran the simulation until the system is in the thermal equilibrium state. Then, we connected the pseudo beads lying nearby on different polymer chains to form the network, corresponding to a gelation process in the experiments. The connection process continues until the reaction ratio becomes 0.9. The correspondence of the slip model of the simulation and the actual SR gels is shown in Figure 1 (bottom). We did not introduce unreacted CDs in the simulation as much as the experiments, for sake of simplicity. In the case of the non-slip model, we fixed the spring of the pseudo beads to the randomly selected beads on the main chain.

#### 2.1.2. Results

We deformed the network isotropically to determine the equilibrium swelling ratio. Microscopic stress tensor $\tau_{\alpha \beta }$ is described by the relative position vector and the force vector as

(1) $$\begin{array}{c} \tau_{\alpha \beta }=-\frac{1}{V}\sum_{ij}r_{ij}^{\alpha }F_{ij}^{\beta }-\frac{1}{V}\sum_{ij}mv_{i}^{\alpha }v_{j}^{\beta } \end{array}$$

where $v_{i}^{\alpha }$ is a α th component of the velocity vector $dr_{i}/dt$. This stress tensile is the force per unit deformed cross-sectional area. The stress for the isotropic deformation is obtained as average force in the principal directions

(2) $$\begin{array}{c} \tau =(\tau_{xx}+\tau_{yy}+\tau_{zz})/3. \end{array}$$

Swelling ratio is defined as $q\equiv V/V_{0}$ where *V* is the volume when the network is deformed and $V_{0}$ is the initial volume when the network is formed. Equilibrium swelling ratio $q_{\mathrm{eq}}$ is determined as *q* at τ=0.

**Figure 2:**
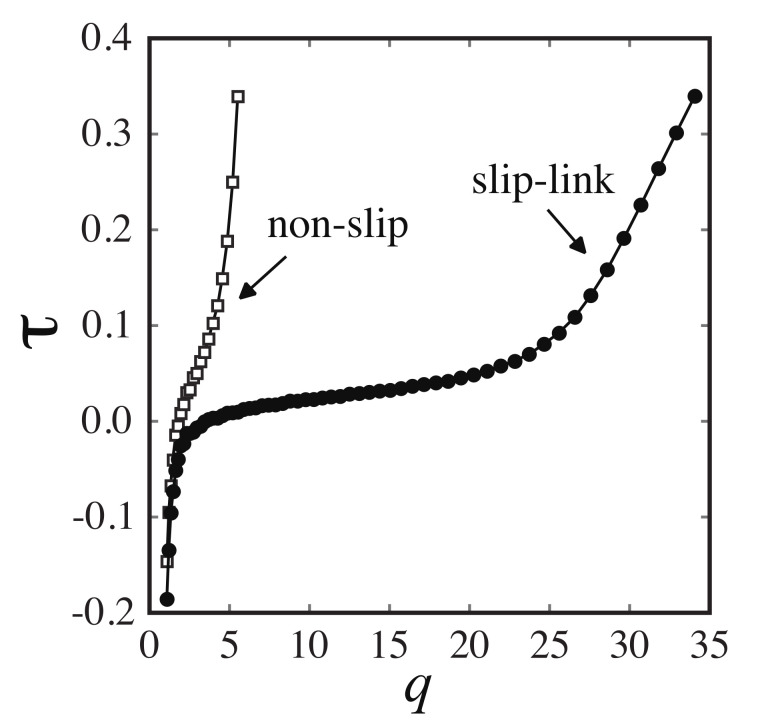
Stress τ as a function of the swelling ratio *q* for the slip model (•) and the non-slip model (□), calculated by the molecular simulations.

We present the stress τ as a function of the swelling ratio *q* in Figure 2. The equilibrium swelling ratios for the non-slip model and the slip model were determined as $q_{\mathrm{eq}}^{\mathrm{non}-\mathrm{slip}}=3.1$ and $q_{\mathrm{eq}}^{\mathrm{slip}}=5.6$, respectively. In the experiments, the network is deswelled from the equilibrium state [20]. Likewise, in our simulations, the S–S relationships are calculated in the deswelled state ($q^{\mathrm{non}-\mathrm{slip}}=1.1$ and $q^{\mathrm{slip}}=1.2$). The number densities of the simulation for the slip and non-slip model are $0.3\sigma^{-3}$ and $0.33\sigma^{-3}$, respectively.

**Figure 3:**
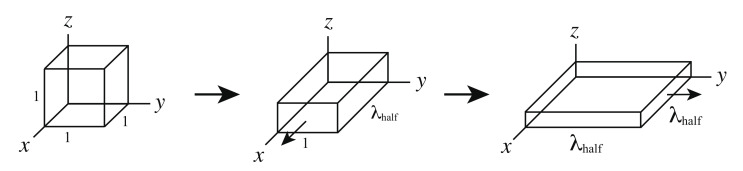
Schematic figure of the two steps biaxial deformation.

After the swelling ratios are determined, we calculate the stresses under two steps biaxial deformation. Two steps biaxial deformation is divided into two steps as shown in Figure 3. In the first step, the network is deformed in the *x*-direction until $\lambda_{x}=\lambda_{\mathrm{half}}$ with $\lambda_{y}$ fixed at 1. In the second step, the network is deformed in the *y*-direction until $\lambda_{y}=\lambda_{\mathrm{half}}$ with $\lambda_{x}$ fixed at $\lambda_{\mathrm{half}}$, where $\lambda_{x}$ and $\lambda_{y}$ are the stretch ratios of *x* and *y* axes respectively. The stresses in the x− and y−directions under the biaxial deformation are $\sigma_{x}$ and $\sigma_{y}$, respectively.

**Figure 4:**
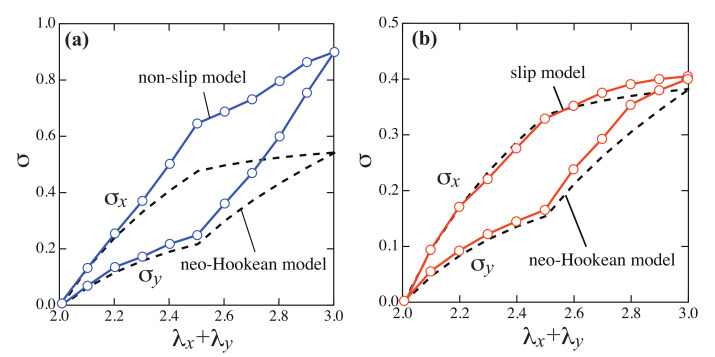
S–S curves in two-steps biaxial deformation for (**a**) the non-slip model and (**b**) the slip model, calculated by the molecular simulations. The S–S curves of the NH model are shown as dashed lines.

Figure 4 shows the stresses $\sigma_{x}$ and $\sigma_{y}$ as a function of the sum of the stretch ratios $\lambda_{x}+\lambda_{y}$. For comparison, the stresses of the NH model normalized by the initial modulus of elasticity are shown as dotted lines in Figure 4. NH model is a simple rubber elasticity model for ideal chains, and its elastic free energy is written as

(3) $$\begin{array}{c} F=\frac{G}{2}\left(\lambda_{x}^{2}+\lambda_{y}^{2}+\lambda_{z}^{2}\right) \end{array}$$

where *G* is an elastic modulus, $\lambda_{i}$, is a stretch ratio of axis *i*. The stress tensors of *x* and *y* axes in general biaxial deformations are expressed by

(4) $$\begin{array}{c} \sigma_{x}=G\left(\lambda_{x}-\frac{1}{\lambda_{x}^{3}\lambda_{y}^{2}}\right), \end{array}$$

(5) $$\begin{array}{c} \sigma_{y}=G\left(\lambda_{y}-\frac{1}{\lambda_{y}^{3}\lambda_{x}^{2}}\right). \end{array}$$

The stresses of the non-slip model deviate upward from NH model overall. On the other hand, the stresses of the slip model are in good agreement with NH model. The stresses for the non-slip and the slip model obtained by molecular simulations exhibited qualitatively same behaviors as the experimental results. In the non-slip model, the stress on the network can not be relaxed because of the fixed cross-links and the stress became large when the network is largely deformed. In the slip model, the stress is relaxed by slip-links varying the network structure, hence, the stress is smaller than that of the non-slip model.

Here we discuss the chain length of the actual gels in the experiments, especially the subchain length and the corresponding number of beads in the coarse-grained bead-spring model, which is directly related to the elastic modulus. The subchain length of SR gels, i.e., the number of monomer units between cross-linked CDs, is calculated as 23 [20]. The corresponding number $n_{\mathrm{sub}}$ of beads in the coarse-grained model is about 12 if we take the Kuhn length as a coarse-graining length scale [25]. For the chemical gels used in the experiments, we estimated the subchain length from the elastic modulus. Using the relationship G=ρRT/M, where ρ is the density, *M* is the molecular weight of subchain, we obtain the average molecular weight of a subchain M≃5071g/mol [19], which corresponds to the number of monomers for a subchain $N_{\mathrm{sub}}≃94$ and the number of beads for a subchain in the coarse-grained model $n_{\mathrm{sub}}≃54$, respectively.

Although the estimated value of $n_{\mathrm{sub}}$ in both SR and chemical gels, is several times larger than that in the simulation ($n_{\mathrm{sub}}≃4$), the effective subchain length for both gels is expected to be smaller than the estimated ones. In the case of the SR gels used in the measurements are swollen from the prepared state, the effective subchain length is expected to be smaller than the estimated value. In the case of chemical gels, there are many subchains much shorter than average length in networks due to the randomness of the reaction [26]. In fact, the nonlinear stretching effect observed in the experiment, i.e., the deviation of the S–S curves of chemical gels from that of the NH model, is expected to be caused by such shorter subchains rather than the average-sized subchains. Therefore, it is expected that the behavior of the non-slip model in Figure 4 is closely related to the nonlinear stretching effect of the chain. This will be studied in the next section in detail.

It is also noted that the subchain length of our simulation model is shorter than the entanglement length [24]. This is consistent with the experimental situation where the entanglement effects are not significant.

### 2.2. Tetrahedral Model with Gaussian Chain

**Figure 5:**
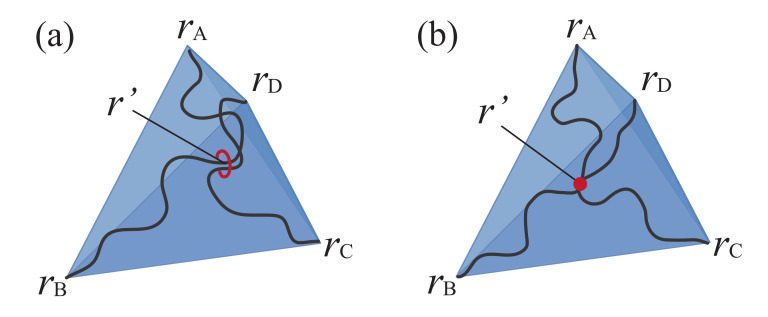
Schematic figures of the tetrahedral model for (**a**) the slip model and (**b**) the non-slip model.

**Figure 6:**
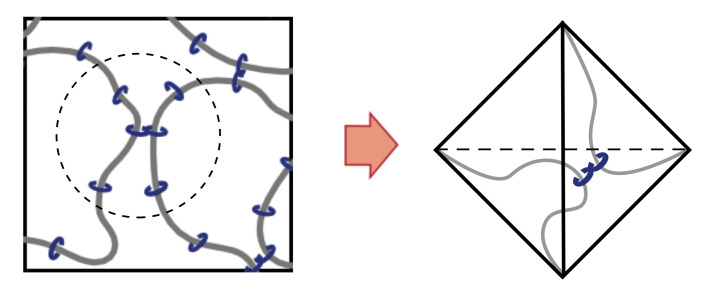
The correspondence between the local part of SR gels (**left**) and the tetrahedral slip model (**right**).

To investigate the effect of slip-links in biaxial deformation in more detail, we introduce the theoretical models for the slip model. Pearson and Graessley have introduced the Flory–Rehner type tetrahedral model which incorporates the slip-link to describe the chain entanglement effect on rubber elasticity [27]. This model is composed of two chains having *n* segments with its both ends fixed at the vertices of the tetrahedron. The chains intersect at one cross-linking point which can be anywhere in the space and along the chain, i.e., the cross-link can slide along the chains (Figure 5a). Since this model can be regarded as a model of movable cross-links in SR gels, we employed this model to study the elasticity of SR gels. The correspondence of the SR gels and the tetrahedral slip model is shown in Figure 6. We extract the local part around the cross-linking point of SR gels as the tetrahedron. We compared the stress of two types of models: the slip model and the non-slip model where the two chains intersect at the middle point of the chains (Figure 5b).

#### 2.2.1. Model

Since the tetrahedral non-slip model is identical to the Flory–Rehner tetrahedral model, the stress of the non-slip model can be obtained by using this theory [28]. In the following, we show the calculation of the stress for the tetrahedral slip model under biaxial deformation.

We calculated the free energy in the deformed state by counting the number of possible configurations. Instead of considering all possible random orientations of tetrahedron, for the sake of simplicity, we considered only three orientations along the three principal axes. It is expected that averaging with only three directions does not lead to unphysical bias because slightly shifted orientation produces the similar results.

**Figure 7:**
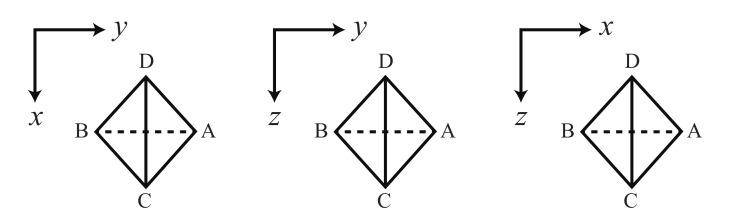
Three orientations of the tetrahedron used to calculate the number of states.

We considered the following three orientations of the tetrahedron (Figure 7).

1. the edge (A, B) and the edge (C, D) are parallel to z and y-axis respectively.
2. the edge (A, B) and the edge (C, D) are parallel to z and x-axis respectively.
3. the edge (A, B) and the edge (C, D) are parallel to x and y-axis respectively.

Each vertex of the tetrahedron is denoted as A, B, C and D, and the edge between vertex X and vertex Y is written as (X, Y).

The number of states per unit volume in the unswollen state is ρ, and each ρ/3 is oriented to each direction. In addition, there are three possible chain pairs (AB, CD), (AC, BD), (AD, BC), where the chain is labeled according to the vertices to which the chain is attached. Considering all possible orientations and chain pairs, we have nine cases, each of which is denoted as *m*.

Next, let us consider the entropy of chain configurations. It is assumed that the chain has *n* segments, and the chains intersect at the *i*-th segment on one of the chains and the *j*-th segment on the other chain. The coordinate of the cross-link is $r^{\prime }=\left(x^{\prime },y^{\prime },z^{\prime }\right)$. The number of configurations for all possible states is

(6) $$\begin{array}{c} \Omega =\sum_{i=1}^{n}\sum_{j=1}^{n}{\;\int \int \int \;}_{-\infty }^{\infty }p_{A}p_{B}p_{C}p_{D}dr^{\prime } \end{array}$$

where

$$\begin{array}{ccc} p_{A} & = & {\left(\frac{3}{2\pi ia^{2}}\right)}^{3/2}\exp\left(-\frac{3}{2ia^{2}}{\left|r^{\prime }-r_{A}\right|}^{2}\right),\\ p_{B} & = & {\left(\frac{3}{2\pi \left(n-i\right)a^{2}}\right)}^{3/2}\exp\left(-\frac{3}{2\left(n-i\right)a^{2}}{\left|r^{\prime }-r_{B}\right|}^{2}\right),\\ p_{C} & = & {\left(\frac{3}{2\pi ja^{2}}\right)}^{3/2}\exp\left(-\frac{3}{2ja^{2}}{\left|r^{\prime }-r_{C}\right|}^{2}\right),\\ p_{D} & = & {\left(\frac{3}{2\pi \left(n-j\right)a^{2}}\right)}^{3/2}\exp\left(-\frac{3}{2\left(n-j\right)a^{2}}{\left|r^{\prime }-r_{D}\right|}^{2}\right), \end{array}$$

where *a* is the segment size. The subscript of *p* denotes the chain between vertices A,B,C,D to the cross-link. The vectors $r_{A},r_{B},r_{C}$, and $r_{D}$ are the positions of the vertices of the tetrahedron. The deformation tensor is

(7) $$\begin{array}{c} E=\left[\begin{array}{ccc} \lambda_{x} & 0 & 0\\ 0 & \lambda_{y} & 0\\ 0 & 0 & \lambda_{z} \end{array}\right]. \end{array}$$

The condition of incompressible deformation requires $\lambda_{z}=1/\lambda_{x}\lambda_{y}$. The number of configurations Ω is a function of **E**.

(8) $$\begin{array}{ccc} \Omega (E) & = & \sum_{i=1}^{n}\sum_{j=1}^{n}{\left(\frac{3}{2\pi na^{2}}\right)}^{6}{\left(\frac{n^{4}}{i(n-i)j(n-j)}\right)}^{3/2}\\ & & \times \prod_{k=1}^{3}\int_{-\infty }^{\infty }\exp\left(-\lambda_{k}^{2}\beta^{2}\left(A_{ij}{\left(r_{k}^{\prime }-\frac{B_{kij}}{2A_{ij}}\right)}^{2}-\frac{B_{kij}^{2}}{4A_{ij}}+C_{kij}\right)\right)dr_{k}^{\prime }, \end{array}$$

where $\beta^{2}\equiv 3/2{\langle r^{2}\rangle }_{0}$, ${\langle r^{2}\rangle }_{0}$ is the mean squared end-to-end distance of a free strand, and

(9) $$\begin{array}{ccc} A_{ij} & = & \left(\frac{n}{i}+\frac{n}{n-i}+\frac{n}{j}+\frac{n}{n-j}\right), \end{array}$$

(10) $$\begin{array}{ccc} B_{kij} & = & \left(\frac{n}{i}r_{Ak}+\frac{n}{n-i}r_{Bk}+\frac{n}{j}r_{Ck}+\frac{n}{n-j}r_{Dk}\right), \end{array}$$

(11) $$\begin{array}{ccc} C_{kij} & = & \left(\frac{n}{i}r_{Ak}^{2}+\frac{n}{n-i}r_{Bk}^{2}+\frac{n}{j}r_{Ck}^{2}+\frac{n}{n-j}r_{Dk}^{2}\right). \end{array}$$

Here, $\lambda_{1},\lambda_{2}$, and $\lambda_{3}$ are just the aliases of $\lambda_{x},\lambda_{y}$ and $\lambda_{z}$, respectively. Applying the Gaussian integral to the Equation (8) leads to

(12) $$\begin{array}{c} \Omega \left(E\right)=\Gamma_{0}\prod_{k=1}^{3}\sum_{i=1}^{n}\sum_{j=1}^{n}A_{ij}^{-1/2}\exp\left(-\lambda_{k}^{2}\beta^{2}\left(-\frac{B_{kij}^{2}}{4A_{ij}}+C_{kij}\right)\right), \end{array}$$

where $\Gamma_{0}$ is the normalization constant given by

(13) $$\begin{array}{c} \Gamma_{0}=\pi^{3/2}{\left(\frac{3}{2\pi na^{2}}\right)}^{6}. \end{array}$$

The edge length of the tetrahedron is assumed to be 2l, and it is equivalent to the root-mean-squared end-to-end distance of the chain $\sqrt{{\langle r^{2}\rangle }_{0}}$, which leads to the relationship $\beta^{2}l^{2}=\frac{3}{8}q^{2/3}$ where *q* is the swelling ratio. Then, Ω is rewritten as

(14) $$\begin{array}{c} \Omega \left(E\right)=\Gamma_{0}\prod_{k=1}^{3}\sum_{i=1}^{n}\sum_{j=1}^{n}A_{ij}^{-1/2}\psi_{kij} \end{array}$$

where

(15) $$\begin{array}{cc} \psi_{kij}= & \exp\left(-\frac{3}{8}q^{2/3}\lambda_{k}^{2}\left(-\frac{B_{kij}^{2}}{4A_{ij}}+C_{kij}\right)/l^{2}\right). \end{array}$$

Since there are ρ/q independent tetrahedra per unit volume, we have (ρ/q)/9 tetrahedra for each orientation and the pair of the strands. Then, the number of configurations for all possible states is

(16) $$\begin{array}{c} \Omega_{T}={\left(\prod_{m=1}^{9}\Omega_{m}\right)}^{\rho /9q}, \end{array}$$

where $\Omega_{m}$ is the number of configurations for case *m*. We obtained the entropy by the Boltzmann equation

(17) $$\begin{array}{c} S=k_{B}\;ln\Omega_{T} \end{array}$$

where $k_{B}$ is the Boltzmann constant. We differentiated the free energy change with respect to the stretch ratio, to obtain the stress as

(18) $$\begin{array}{c} \sigma_{x}\left(E\right)=-k_{B}T\left(\frac{\partial ln\Omega_{T}}{\partial \lambda_{x}}\right)=-\frac{\rho }{9q}k_{B}T\sum_{m=1}^{9}\frac{1}{\Omega_{m}}\frac{\partial \Omega_{m}}{\partial \lambda_{x}}. \end{array}$$

where *T* is the temperature. The stress is calculated as

(19) $$\begin{array}{ccc} & & \sigma_{x}\left(E\right)=-\frac{\rho }{9q}k_{B}T\\ & & \times \sum_{m=1}^{9}\sum_{i=1}^{n}\sum_{j=1}^{n}D_{ij}\left(-\frac{3}{4}q^{2/3}\left(\lambda_{x}\left(-\frac{B_{xij}^{2}}{4A_{xij}}+C_{xij}\right)/l^{2}-\frac{1}{\lambda_{x}^{3}\lambda_{y}^{2}}\left(-\frac{B_{zij}^{2}}{4A_{zij}}+C_{zij}\right)/l^{2}\right)\right)\\ & & \times \exp\left(-\frac{3}{8}q^{2/3}\sum_{k=1}^{3}\lambda_{k}^{2}\left(-\frac{B_{kij}^{2}}{4A_{ij}}+C_{kij}\right)/l^{2}\right)\\ & & /\sum_{i=1}^{n}\sum_{j=1}^{n}D_{ij}\exp\left(-\frac{3}{8}q^{2/3}\sum_{k=1}^{3}\lambda_{k}^{2}\left(-\frac{B_{kij}^{2}}{4A_{ij}}+C_{kij}\right)/l^{2}\right) \end{array}$$

where

(20) $$\begin{array}{c} D_{ij}={\left(\frac{n^{2}}{i(n-i)+j(n-j)}\right)}^{3/2}. \end{array}$$

We can obtain $\sigma_{y}\left(E\right)$ just by interchanging *x* and *y* in Equation (19).

#### 2.2.2. Results

**Figure 8:**
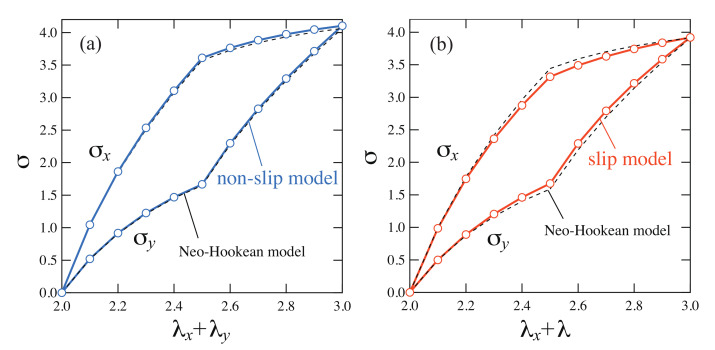
S–S curves in two-steps biaxial deformation for (**a**) the non-slip model and (**b**) the slip model with the Gaussian chain in the tetrahedron model. The S–S curves for the NH model are shown as dashed lines.

The calculated stresses as a function of the stretch ratio for the slip and non-slip model in biaxial deformation are shown in Figure 8. The stresses for the non-slip model coincide with the NH model. This result is trivial because this model is equivalent to the Flory–Rehner model which produces the same S–S curves as NH models. Similarly, the stresses of the slip model are in good agreement with those of the NH model. However, the shape is slightly narrower than the NH model. One can consider that this small difference is attributed to the effect of the slip-link. In fact, this shape is close to that of the slip-link theory of Edwards-Vilgis [13] with the slippage parameter η=0.05. In the tetrahedral model, only a small difference was observed between the slip model and the non-slip model, whereas a distinctive difference was observed in the experiments. The upward deviation from the NH model observed in the simulation and the experiments of chemical gels is expected to be caused by a nonlinear stretching effect due to the finite extensibility of chains. In the case of the Gaussian chain, the chain can be elongated infinitely where the stress is proportional to the stretch ratio, however, in reality we cannot elongate the chain exceeding the contour length of the chain. The large stress is observed as the elongation length gets closer to the contour length. The stress of the nonlinear stretching effect is modeled by the Langevin chain. Therefore, we analyze the tetrahedral model with the Langevin chain in the next section.

### 2.3. Tetrahedral Model with Langevin Chain

#### 2.3.1. Model

In the previous section, we calculated the S–S relationships of the tetrahedral model with Gaussian chain. Here, we introduce the Langevin chain to the tetrahedral model to investigate the effect of nonlinearity of the chain elongation. The S–S relationship of the Langevin chain is expressed by the inverse Langevin function [23]:

(21) $$\begin{array}{c} \frac{af}{k_{B}T}=L^{-1}\left(\frac{R}{na}\right), \end{array}$$

where $af/k_{B}T$ is a nondimensional force which is the ratio of af, an energy required to elongate the chain by a unit length *a*, to the unit of energy $k_{B}T$. Here, $L^{-1}$ is the inverse Langevin function. The Langevin function is defined as

(22) $$\begin{array}{c} L\left(x\right)\equiv \frac{d}{dx}\left\{\ln\left[\frac{\sinh x}{x}\right]\right\}=\coth x-\frac{1}{x}. \end{array}$$

The relationship between the change in free energy dF and the change in end-to-end vector length dR is

(23) $$\begin{array}{c} dF=-SdT+f\;dR. \end{array}$$

In the isothermal condition dT=0, the free energy is obtained by

(24) $$\begin{array}{ccc} F/k_{B}T & = & \int_{0}^{R}fdR/k_{B}T \end{array}$$

(25) $$\begin{array}{ccc} & = & \int_{0}^{R}L^{-1}\left(\frac{y}{na}\right)dy/a. \end{array}$$

Substituting $y^{\prime }=y/na$ into the equation above leads to

(26) $$\begin{array}{ccc} F/k_{B}T & = & n\int_{0}^{R/na}L^{-1}\left(y^{\prime }\right)dy^{\prime } \end{array}$$

(27) $$\begin{array}{ccc} & = & n\left\{\frac{R}{na}L^{-1}\left(\frac{R}{na}\right)+\ln\frac{L^{-1}\left(\frac{R}{na}\right)}{\sinh L^{-1}\left(\frac{R}{na}\right)}\right\}+C. \end{array}$$

Now, let us use the Langevin chain in the tetrahedral model. The edge length of the tetrahedron 2l is equal to the root-mean-squared end-to-end distance of the free Gaussian chain $\sqrt{{\langle r^{2}\rangle }_{0}}$. The number of chain segments is *n*, and the chains intersect at the *i*-th segment on one of the chains and the *j*-th segment on the other chain. The coordinate of the cross-link is $r^{\prime }=\left(x^{\prime },y^{\prime },z^{\prime }\right)$. The free energy per unit volume in the deformed state is

(28) $$\begin{array}{c} \frac{F(i,j)}{k_{B}T}=\frac{3\sqrt{2}}{4}\left(F_{A}+F_{B}+F_{C}+F_{D}\right) \end{array}$$

where $F_{A},\;F_{B},\;F_{C}\;\mathrm{and}\;F_{D}$ are the free energies of the subchains. The subscript of *F* denotes the vertices to which the subchains are attached. The stress $\sigma_{k}$ is obtained as the derivative of the free energy $F\left(i,j\right)/k_{B}T$ with respect to the stretch ratio $\lambda_{k}$, where *k* denotes the stretch direction

(29) $$\begin{array}{ccc} \sigma_{k}\left(i,j,r^{\prime }\right)=\frac{\partial }{\partial \lambda_{k}}\left(\frac{F(i,j)}{k_{B}T}\right) & = & \frac{3\sqrt{2}}{4}\left(\frac{\partial }{\partial \lambda_{k}}F_{A}+\frac{\partial }{\partial \lambda_{k}}F_{B}+\frac{\partial }{\partial \lambda_{k}}F_{C}+\frac{\partial }{\partial \lambda_{k}}F_{D}\right). \end{array}$$

The stress $\sigma_{k}$ is obtained by averaging $\sigma_{k}\left(i,j,r^{\prime }\right)$ with respect to *i*, *j*, and $r^{\prime }$, as

(30) $$\begin{array}{c} \sigma_{k}={\;\int \int \int \;}_{-\infty }^{\infty }\sum_{i=1}^{n}\sum_{j=1}^{n}\sigma_{k}\left(i,j,r^{\prime }\right)p\left(i,j,r^{\prime }\right)dr^{\prime }. \end{array}$$

The normalized probability function $p\left(i,j,r^{\prime }\right)$ is expressed using the partition function *Z* as

(31) $$\begin{array}{c} p\left(i,j,r^{\prime }\right)=\frac{1}{Z}\exp\left(-\frac{F(i,j,r^{\prime })}{k_{B}T}\right) \end{array}$$

where *Z* is given by

(32) $$\begin{array}{c} Z={\;\int \int \int \;}_{-\infty }^{\infty }\sum_{i=1}^{n}\sum_{j=1}^{n}\exp\left(-\frac{F(i,j,r^{\prime })}{k_{B}T}\right)dr^{\prime }. \end{array}$$

The stress of the non-slip model is obtained by fixing the cross-link at the middle point of the chain. Since the integral with respect to $dr^{\prime }$ cannot be calculated analytically, we numerically integrate Equation (30).

#### 2.3.2. Results

**Figure 9:**
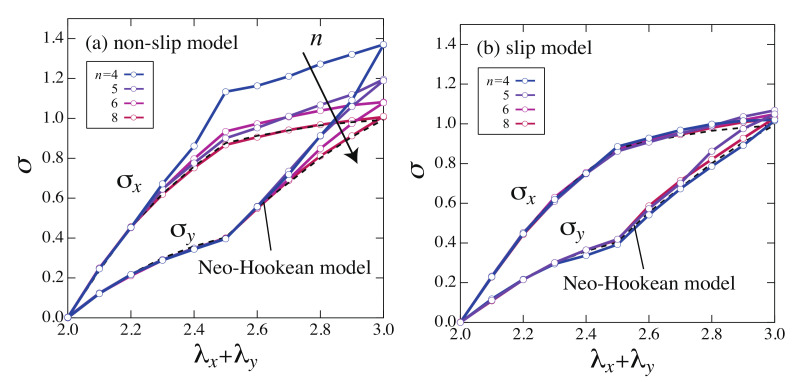
S–S curves in two-steps biaxial deformation for (**a**) the non-slip model and (**b**) the slip model which uses the Langevin chain with various number of chain segments *n*. The S–S curves for the NH model are shown as dashed lines.

We calculated the stress of the tetrahedral model with the Langevin chain as a function of the stretch ratio (Figure 9). In the non-slip model, as the number of segments *n* decreases, the nonlinear stretching effect of chain is more pronounced, and the shape of the stress deviates more upward from the NH model. The S–S curves for n=8 are close to those of the NH model since the nonlinear stretching effect of chain is relatively small. In the slip model, the S–S relationships are in good agreement with those of the NH model regardless of the number of segments *n*. This result suggests that the slip-link suppresses the nonlinear stretching effects by moving.

A distinctive difference between the slip and the non-slip model was observed in the Langevin chain models, especially when the number of segments *n* is small, although a little difference was observed in the Gaussian chain models. The difference between chemical gels and SR gels observed in the experiments is explained well by the Langevin chain model.

The segment length n=4 seems too small compared with the subchain length of actual chemical gels. However, as discussed in Section 2.1.2, in the case of chemical gels, there are many subchains much shorter than average length in networks due to the randomness of the reaction, and the deviation of the S–S curves of chemical gels from that of NH model is expected to be caused by the nonlinear stretching effect of such shorter subchains [26].

Furthermore, the nonlinear stretching effect is more pronounced under biaxial deformation than the uniaxial deformation in experiments. This is because, in uniaxial deformation, only one direction is constrained, and the other directions are not constrained, whereas in the biaxial deformation, two directions are constrained where the degree of freedom of the network is significantly suppressed.

On the other hand, in SR gels, the stress is not concentrated on the shortest chain by the effect of slip-links, even in the biaxial deformation. Therefore, the stress of SR gels exhibits good agreement with that of the NH model.

## 3. Conclusions

In this paper, we explored the influence of sliding cross-links on the elastic properties of SR gels under biaxial deformation computationally and theoretically. In the simulations, we compared the S–S relationships between the slip model where the network has movable cross-links, and the non-slip model where the network is permanently fixed by the cross-links. We found that the S–S curves of the non-slip model deviate upward from the NH model, while those of the slip model are close to the NH model. This result qualitatively agrees with the experimental results. In the theoretical analysis, we examined the effect of slip-links on the S–S behavior in more detail by using Flory–Rehner type tetrahedral model. In this model, we considered two types of chains, the Gaussian chain and the Langevin chain, to examine the nonlinear stretching effect of the chains. We found that the non-slip model with the Gaussian chain is equivalent to the NH model, while the slip model with the Gaussian chain exhibits slightly narrower shape, however, almost agrees with the NH model. The stress of the non-slip model with the Langevin chain exhibits large deviation from the NH model when the segment length is small. The stress of the slip model with the Langevin chain is close to the NH model regardless of the number of segments. This result suggests that the slip-link allows the network to avoid the largely stressed situation, as a result, the S–S relationships of SR gels are well described by the NH model. This molecular picture explains the results observed in both molecular simulations and experiments. To study S–S behaviors of SR gels and chemical gels in more detail, we may need to consider other nonlinearities such as the constraint of the thermal fluctuation of chains and cross-linking junctions. However, the peculiar elastic behaviors of SR gels observed in the measurements under biaxial deformation are well explained by suppressing the nonlinear chain-stretching effect by sliding of the cross-links.

The coarse-grained description of the figure-of-eight cross-links using simple bonds [15] or pseudo beads in the present study is quite effective to study the physical properties of the SR gels. By using this method, we will study molecular mechanism of fascinating physical properties of SR gels observed by experiments, e.g., effects of unreacted cyclodextrins between the figure-of-eight cross-links on elastic properties of SR gels and peculiar solvent permeability of SR gels [29], in future publications.

## 4. Methods

### Molecular Simulation

In our simulations, the elastic force between connected beads is described by a finitely extensible nonlinear elastic (FENE) potential:

(33) $$\begin{array}{c} u_{b}\left(l\right)=\left\{\begin{array}{cc} -\frac{1}{2}k_{b}\Delta l_{m}^{2}\ln\left[1-{\left(\frac{\Delta l_{i}}{\Delta l_{m}}\right)}^{2}\right] & (|\Delta l_{i}|<\Delta l_{m})\\ \infty & (|\Delta l_{i}\left|\geq \Delta l_{m}\right) \end{array}\right. \end{array}$$

where $k_{b}$ is the spring constant, $l_{i}$ is the length of bond *i*, $l_{\max}$ is the maximum bond length, $l_{0}$ is the equilibrium bond length, and $\Delta l_{i}\equiv l_{i}-l_{0},\;\Delta l_{m}\equiv l_{\max}-l_{0}$. Non-bonded beads are assumed to interact via the following purely repulsive Lennard–Jones(LJ) potential:

(34) $$\begin{array}{c} u_{\mathrm{LJ}}\left(r\right)=\left\{\begin{array}{cc} 4\epsilon \left[{\left(\frac{\sigma }{r}\right)}^{12}-{\left(\frac{\sigma }{r}\right)}^{6}+\frac{1}{4}\right] & \left(r\leq 2^{\frac{1}{6}}\sigma \right)\\ 0 & \left(r>2^{\frac{1}{6}}\sigma \right) \end{array}\right. \end{array}$$

where σ and ϵ are the unit length and the unit energy of the LJ potential, respectively. The parameters are chosen as follows: $k_{b}=1500\epsilon /\sigma^{2}$, $l_{\max}=1.2\sigma$ and $l_{0}=1.0\sigma$. The equation of motion of the bead *i* is given by the following Langevin equation:

(35) $$\begin{array}{c} m\frac{d^{2}r_{i}\left(t\right)}{dt^{2}}=F_{i}-\zeta \frac{d}{dt}r_{i}\left(t\right)+R_{i}\left(t\right) \end{array}$$

where *m* being the mass of beads, $r_{i}$ the position vector of the bead *i*, and ζ the friction coefficient. The force on the bead *i* is $F=-\partial U_{i}/\partial r$ where $U_{i}$ is the sum of all potentials of the bead *i*. The fluctuation force $R_{i}$ satisfies the fluctuation-dissipation theorem. We solved Langevin equation by using the velocity Verlet method [30]. In the simulation, we calculated the stress for the slip model where the pseudo beads can slip along the chain and the non-slip model where the pseudo beads are fixed at the specific point on the polymer chains.
